# Supplementary material for: A Likelihood Approach for Real-Time Calibration of Stochastic Compartmental Epidemic Models
Source: PLoS Comput Biol. 2017 Jan 17;13(1):e1005257. doi: 10.1371/journal.pcbi.1005257 (PMC5240920; doi:10.1371/journal.pcbi.1005257)
Supplement: S1 File — (TAR.GZ) [file pcbi.1005257.s014.tar.gz › HSPH_Online-SI-Revision/EnKF07/n100-mild/n100-mild_EnKF_table8.pdf]

1	R0	{1.24125, 3.38982}	{2.73721, 4.4678}	{{2.27554, 2.07454}, {3., 9.17427}}	{120.521, 74.3388}	inf	161.	7999.63	{2716.43, 11 586.8}	4870.41	1.50166	newly	287.	9237.58	{1652., 34 692.}	3121.21	1.28817	Reff	0.815836	0.465909	{0.139876, 0.913665}	59.7918	ar	34 926.	186 434.	{163 857., 230 887.}	433.798
2	R0	{1.29554, 4.51878}	{2.16302, 5.83829}	{{0.3426, 0.278559}, {3., 15.4766}}	{105.123, 101.569}	inf	262.	5003.3	{3604.59, 3604.59}	1811.4	1.13443	newly	367.	4309.78	{1359., 6459.}	1077.32	0.939706	Reff	0.75267	0.308135	{0.0698275, 0.555425}	64.4452	ar	42 828.	175 690.	{159 982., 215 025.}	310.221
3	R0	{1.2443, 3.64292}	{2.46545, 10.6711}	{{1.47687, 6.57242}, {3., 14.4929}}	{98.1394, 192.928}	inf	255.	6209.34	{1765.91, 21 528.3}	2335.04	1.00263	newly	387.	3366.44	{740., 12 617.}	769.881	0.591448	Reff	0.790168	0.545588	{0.284692, 1.4755}	51.962	ar	37 087.	175 872.	{158 260., 260 048.}	374.215
4	R0	{1.38465, 3.43331}	{2.48151, 10.6348}	{{1.55864, 2.53767}, {3., 13.9593}}	{79.2155, 222.801}	inf	64.	2697.1	{102.245, 2822.21}	4120.39	1.43281	newly	86.	1926.42	{42., 3930.}	2159.49	1.19939	Reff	0.700356	0.439041	{0.0292176, 0.814591}	53.5446	ar	49 511.	167 683.	{156 062., 174 897.}	238.679
5	R0	{1.25409, 4.1068}	{2.51276, 10.017}	{{1.51003, 6.57588}, {3., 14.4178}}	{100.365, 143.914}	inf	297.	2362.31	{1440.66, 1440.66}	697.075	0.732098	newly	386.	1451.19	{614., 1320.}	277.469	0.42564	Reff	0.780997	0.649307	{0.312747, 1.00321}	36.395	ar	38 571.	166 234.	{157 140., 170 194.}	330.981
6	R0	{1.28821, 4.2016}	{2.75172, 7.00902}	{{2.13376, 3.17662}, {3., 10.0202}}	{114.221, 88.2855}	inf	242.	7285.6	{901.549, 9594.62}	2912.16	1.31756	newly	315.	8444.37	{1022., 24 811.}	2582.58	1.1757	Reff	0.761538	0.572625	{0.143123, 1.47575}	64.153	ar	41 468.	182 071.	{162 095., 247 251.}	339.064
7	R0	{1.24369, 2.56007}	{2.53144, 13.4966}	{{1.16889, 6.42395}, {3., 17.5917}}	{106.172, 427.197}	inf	25.	2607.23	{255.297, 5611.03}	10 328.9	1.70672	newly	53.	1574.64	{105., 4349.}	2871.74	1.0885	Reff	0.764869	0.638229	{0.0499663, 1.16754}	40.7712	ar	38 603.	168 327.	{157 355., 201 650.}	336.046
8	R0	{1.232, 3.53942}	{2.55652, 6.51545}	{{1.70395, 3.8161}, {3., 12.2546}}	{107.51, 84.0825}	inf	101.	8130.19	{3670.7, 16 269.9}	7949.69	1.67874	newly	163.	6439.66	{1377., 11 476.}	3850.71	1.40406	Reff	0.809486	0.479382	{0.18001, 0.574482}	49.142	ar	34 555.	180 896.	{166 332., 206 453.}	423.501
9	R0	{1.36262, 3.85631}	{2.70347, 14.69}	{{2.13712, 3.84142}, {3., 19.0725}}	{98.4023, 281.087}	inf	139.	8891.46	{957.057, 10 368.1}	6296.87	1.51537	newly	200.	4761.98	{408., 7647.}	2282.76	1.07388	Reff	0.708181	0.819125	{0.0281986, 2.40708}	87.9279	ar	48 410.	188 019.	{157 516., 279 357.}	288.388
10	R0	{1.28702, 4.67968}	{2.4036, 4.17276}	{{1.3202, 2.76046}, {3., 5.51392}}	{98.2603, 26.5199}	inf	267.	7796.21	{3598.51, 3598.51}	2819.93	1.19183	newly	358.	10 080.4	{2710., 5154.}	2715.74	1.05226	Reff	0.767643	0.319755	{0.0249052, 0.528255}	77.1681	ar	41 092.	175 970.	{162 123., 200 610.}	328.233
11	R0	{1.35752, 3.47129}	{2.47118, 10.22}	{{1.51381, 6.70561}, {3., 14.9325}}	{82.0365, 194.415}	inf	143.	5065.96	{2637.28, 2637.28}	3442.63	1.30585	newly	225.	2845.18	{1088., 2285.}	1164.52	0.90115	Reff	0.755256	0.60197	{0.269247, 1.05533}	45.9666	ar	44 669.	175 791.	{161 663., 232 983.}	293.542
12	R0	{1.35929, 5.83647}	{2.16637, 2.95127}	{{0.390944, 0.931291}, {3., 6.70903}}	{104.782, 56.909}	inf	598.	6099.79	{3233.02, 3233.02}	920.032	0.802925	newly	613.	8006.9	{2477., 10 302.}	1206.18	0.896244	Reff	0.753917	0.458581	{0.0607739, 0.924202}	55.3272	ar	46 715.	172 642.	{159 531., 192 818.}	269.565
13	R0	{1.28019, 3.29908}	{2.73657, 6.29319}	{{2.16331, 3.89525}, {3., 8.67745}}	{113.763, 90.7559}	inf	135.	4903.02	{3232.25, 5326.52}	3531.86	1.50677	newly	244.	5733.17	{2124., 10 356.}	2250.89	1.23542	Reff	0.759998	0.638707	{0.18994, 2.16329}	56.5811	ar	40 941.	182 126.	{160 350., 249 396.}	344.85
14	R0	{1.36661, 3.48318}	{2.26213, 6.32243}	{{0.616401, 3.59928}, {3., 9.78949}}	{99.4107, 81.5131}	inf	77.	4003.73	{372.259, 3856.67}	5107.01	1.38269	newly	115.	3133.87	{353., 8333.}	2633.87	1.14581	Reff	0.705239	0.372555	{0.0448293, 0.684446}	50.0634	ar	48 672.	165 735.	{154 794., 216 912.}	240.513
15	R0	{1.35865, 3.84123}	{2.54627, 9.8724}	{{1.5781, 5.09383}, {3., 13.8495}}	{87.412, 157.011}	inf	163.	5576.54	{797.413, 2545.49}	3321.19	1.19389	newly	228.	3481.86	{378., 2824.}	1429.38	0.881985	Reff	0.704243	0.664043	{0.322148, 0.875464}	41.3891	ar	48 598.	178 117.	{157 808., 232 691.}	266.511
16	R0	{1.22879, 3.98329}	{2.16177, 2.5118}	{{0.453077, 1.02655}, {3., 6.61999}}	{117.224, 60.7715}	inf	367.	3803.03	{3589.62, 3589.62}	936.247	0.957124	newly	573.	4582.95	{2439., 5240.}	699.817	0.837184	Reff	0.822699	0.315703	{0.101693, 0.73998}	61.6259	ar	34 405.	166 312.	{163 402., 170 086.}	383.395
17	R0	{1.34925, 3.64524}	{2.81277, 12.3137}	{{2.4244, 0.7526}, {3., 17.6709}}	{108.469, 277.476}	inf	162.	6346.14	{817.088, 7640.1}	3817.37	1.31025	newly	237.	10 287.8	{299., 23 012.}	4241.99	1.01721	Reff	0.721606	0.828371	{0.021193, 2.42438}	83.6051	ar	46 871.	186 658.	{159 592., 256 690.}	298.238
18	R0	{1.33054, 2.25275}	{2.5948, 7.0832}	{{2.03523, 5.23961}, {3., 7.54578}}	{95.0184, 214.424}	inf	9.	3609.34	{924.943, 2606.16}	40 003.7	2.35117	newly	21.	3322.31	{641., 2860.}	15 720.5	1.89425	Reff	0.740885	0.578619	{0.266803, 1.17369}	56.4312	ar	44 336.	170 536.	{157 904., 184 268.}	284.644
19	R0	{1.28394, 3.69859}	{2.42545, 5.25996}	{{1.44552, 0.35311}, {3., 15.5473}}	{88.9071, 108.373}	inf	204.	1872.27	{1040.39, 1821.73}	819.702	0.829483	newly	360.	3357.85	{457., 3738.}	839.397	0.688356	Reff	0.786067	0.524437	{0.146687, 1.12965}	52.6511	ar	39 558.	166 264.	{155 787., 191 645.}	320.305
20	R0	{1.2883, 2.26493}	{2.51903, 7.16446}	{{2.04723, 5.38743}, {3., 7.93462}}	{95.5313, 216.321}	inf	22.	4412.82	{848.128, 2302.5}	19 959.4	1.99882	newly	37.	4173.55	{590., 6036.}	11 181.6	1.68601	Reff	0.763434	0.535337	{0.13116, 1.76682}	72.0492	ar	40 768.	176 383.	{159 411., 240 293.}	332.651
21	R0	{1.34098, 2.78621}	{2.61307, 4.31486}	{{2.04263, 0.391635}, {3., 9.47744}}	{94.8625, 140.786}	inf	39.	1661.32	{1074.59, 1074.59}	4160.74	1.43665	newly	64.	2911.55	{622., 2900.}	4450.58	1.34762	Reff	0.744378	0.607068	{0.349059, 1.05333}	42.7441	ar	44 605.	167 225.	{159 666., 173 130.}	274.901
22	R0	{1.33783, 5.95911}	{2.47929, 2.88895}	{{1.18544, 1.40471}, {3., 6.86283}}	{89.7123, 58.1933}	inf	686.	4099.19	{1910.49, 2868.66}	506.58	0.700163	newly	709.	7105.62	{1838., 7571.}	910.553	0.760272	Reff	0.760757	0.409679	{0.0621765, 0.783258}	68.4378	ar	45 418.	171 479.	{160 804., 175 536.}	277.556
23	R0	{1.33359, 3.3069}	{2.77225, 8.24813}	{{2.1123, 3.18443}, {3., 11.6991}}	{107.879, 150.533}	inf	84.	9043.7	{2950.2, 10 195.3}	10 666.8	1.7899	newly	143.	8377.68	{2429., 23 737.}	5760.36	1.4668	Reff	0.719592	0.629077	{0.111378, 1.89195}	69.8157	ar	46 245.	180 547.	{160 017., 253 144.}	290.415
24	R0	{1.30588, 2.56123}	{2.44133, 6.95706}	{{1.40361, 3.55737}, {3., 9.01346}}	{86.9489, 177.095}	inf	11.	4896.98	{4015.93, 4015.93}	44 418.	2.60295	newly	29.	5337.8	{2219., 5115.}	18 306.2	2.07971	Reff	0.752997	0.391955	{0.0318267, 0.58544}	62.4867	ar	42 383.	170 294.	{159 874., 215 131.}	301.797
25	R0	{1.29813, 4.51849}	{2.13099, 2.96542}	{{0.355517, 1.65741}, {3., 6.6345}}	{111.518, 53.1033}	inf	473.	4720.7	{4308.33, 4308.33}	898.034	0.952123	newly	633.	5349.26	{2901., 6028.}	745.065	0.866362	Reff	0.756044	0.263943	{0.0734485, 0.411389}	65.0889	ar	43 371.	171 462.	{168 211., 173 407.}	295.337
26	R0	{1.33231, 3.99953}	{2.66327, 4.42425}	{{2.11388, 1.75596}, {3., 9.24823}}	{99.8989, 70.6939}	inf	227.	4755.42	{752.904, 3848.16}	1997.5	1.1216	newly	332.	5014.21	{512., 7867.}	1415.2	1.00697	Reff	0.747852	0.522816	{0.176223, 1.15371}	59.697	ar	44 733.	176 570.	{160 524., 189 259.}	294.719
27	R0	{1.30555, 4.61445}	{2.17463, 3.57988}	{{0.41466, 2.18239}, {3., 6.14936}}	{109.944, 41.7128}	inf	364.	6060.26	{2965.95, 2965.95}	1564.91	0.966263	newly	467.	6522.2	{2102., 4115.}	1296.62	0.866226	Reff	0.764165	0.262852	{0.0459396, 0.405101}	68.5423	ar	42 624.	163 817.	{155 154., 161 514.}	284.331
28	R0	{1.26819, 5.34969}	{2.48521, 5.38642}	{{1.72392, 0.156556}, {3., 14.4629}}	{95.9649, 101.471}	inf	578.	4456.03	{639.378, 3142.47}	681.422	0.604945	newly	683.	6606.59	{514., 23 898.}	881.023	0.613694	Reff	0.806442	0.473917	{0.182317, 1.13423}	56.8681	ar	38 946.	172 018.	{156 886., 186 200.}	341.684
29	R0	{1.19925, 3.00394}	{2.82287, 9.48147}	{{2.47904, 2.56441}, {3., 18.331}}	{135.386, 231.398}	inf	160.	5772.38	{513.172, 9487.57}	3507.74	1.36291	newly	328.	4283.35	{552., 5886.}	1205.9	0.957244	Reff	0.835841	0.72328	{0.153992, 1.508}	54.8153	ar	31 727.	178 740.	{158 083., 215 832.}	463.368
30	R0	{1.35749, 3.75483}	{2.74049, 10.4875}	{{2.05871, 3.21117}, {3., 19.424}}	{101.88, 186.837}	inf	77.	6457.69	{880.289, 5722.62}	8286.61	1.6652	newly	120.	6549.21	{742., 7470.}	5357.67	1.30472	Reff	0.704592	0.605355	{0.0756275, 1.46227}	78.744	ar	48 244.	177 066.	{157 025., 250 281.}	267.021
31	R0	{1.27131, 4.2161}	{2.65812, 4.61181}	{{2.076, 1.85903}, {3., 9.69574}}	{109.085, 71.1305}	inf	338.	8271.67	{821.195, 30 010.4}	2350.94	1.04316	newly	482.	9872.53	{1054., 23 901.}	1954.25	1.0133	Reff	0.797836	0.635695	{0.0212737, 1.46177}	65.1288	ar	38 299.	185 996.	{158 756., 239 444.}	385.643
32	R0	{1.31827, 6.18119}	{2.43063, 2.85758}	{{0.889039, 1.32814}, {3., 6.5258}}	{100.612, 55.9999}	inf	827.	5210.73	{2760.05, 2760.05}	535.271	0.580479	newly	844.	8133.49	{1900., 4910.}	868.226	0.638715	Reff	0.757649	0.38439	{0.0973875, 0.497009}	70.1037	ar	45 262.	165 985.	{155 750., 170 014.}	266.721
33	R0	{1.20398, 1.35167}	{2.61495, 7.33082}	{{2.07999, 5.35517}, {3., 8.00058}}	{117.193, 442.353}	inf	3.	3886.56	{532.345, 2290.13}	129 452.	2.75939	newly	6.	3521.21	{356., 3866.}	58 586.8	2.3599	Reff	0.802742	0.558822	{0.0471823, 1.43864}	66.0607	ar	33 329.	175 270.	{160 618., 194 859.}	425.877
34	R0	{1.32776, 3.146}	{2.79012, 13.3721}	{{2.40637, 3.81456}, {3., 19.0961}}	{110.138, 325.051}	inf	21.	5779.07	{1327.79, 5460.91}	27 419.4	2.26431	newly	25.	3325.89	{743., 7761.}	13 203.6	1.93808	Reff	0.733149	0.778419	{0.222751, 2.40635}	61.4717	ar	44 872.	179 831.	{158 333., 244 217.}	300.765
35	R0	{1.279, 1.11336}	{2.57988, 6.04689}	{{2.05645, 1.11889}, {3., 11.4951}}	{101.711, 443.121}	inf	0.1	4634.98	{222.638, 2626.31}	4.63488×10^{6}	4.28026	newly	0.1	7997.38	{112., 7276.}	7.99728×10^{6}	4.27856	Reff	0.754188	0.490615	{0.00523204, 1.06938}	74.6661	ar	41 033.	166 108.	{150 114., 231 929.}	304.815
36	R0	{1.20029, 1.94717}	{2.56829, 7.35007}	{{2.13035, 5.57853}, {3., 8.41883}}	{113.972, 277.475}	inf	45.	3377.03	{240.393, 8731.38}	7404.77	1.40894	newly	117.	3590.91	{140., 10 075.}	2977.53	0.946874	Reff	0.807351	0.544573	{0.204583, 1.18077}	68.0653	ar	33 234.	174 112.	{156 198., 223 021.}	423.898
37	R0	{1.33216, 3.69685}	{2.79651, 9.93785}	{{2.29829, 2.61097}, {3., 19.3491}}	{109.923, 201.42}	inf	150.	8759.55	{8674.63, 8674.63}	5739.7	1.76512	newly	219.	8969.66	{2850., 13 038.}	3995.74	1.48939	Reff	0.735299	0.703533	{0.337764, 1.75474}	60.9555	ar	45 235.	184 255.	{166 740., 226 402.}	307.329
38	R0	{1.25434, 2.04314}	{1.96939, 6.8141}	{{0.214138, 5.32658}, {3., 7.21336}}	{118.348, 233.511}	inf	2.	3373.16	{77.4623, 4104.03}	168 558.	2.67703	newly	6.	2867.3	{59., 6136.}	47 688.3	2.08256	Reff	0.769437	0.374944	{0.0316843, 0.644988}	63.2657	ar	38 662.	175 316.	{166 602., 183 523.}	353.459
39	R0	{1.27466, 3.0741}	{2.30632, 6.69093}	{{0.884305, 3.49765}, {3., 10.1503}}	{100.489, 117.655}	inf	79.	5309.55	{1390.48, 5449.81}	6620.95	1.59514	newly	134.	5912.19	{1478., 7062.}	4312.08	1.31976	Reff	0.765178	0.576572	{0.166267, 0.882387}	29.9748	ar	40 181.	170 868.	{158 442., 182 654.}	325.246
40	R0	{1.33045, 2.68154}	{2.3891, 7.27983}	{{1.39205, 3.33939}, {3., 8.39952}}	{79.571, 171.479}	inf	10.	5172.9	{3827.65, 3827.65}	51 629.	2.60942	newly	17.	5023.48	{2279., 4252.}	29 449.9	2.2591	Reff	0.719255	0.426713	{0.129282, 0.664042}	58.0775	ar	45 951.	169 582.	{157 610., 187 960.}	269.05
41	R0	{1.37003, 2.83984}	{2.54694, 4.71078}	{{2.0301, 0.368251}, {3., 5.53977}}	{85.9038, 93.738}	inf	53.	3686.03	{192.341, 2219.13}	6854.77	1.36963	newly	99.	9035.3	{167., 11 782.}	9026.57	1.15854	Reff	0.701168	0.569123	{0.2917, 1.20081}	55.7229	ar	48 926.	172 853.	{158 451., 174 091.}	253.296
42	R0	{1.3003, 2.94885}	{2.28263, 6.23043}	{{0.838088, 3.51544}, {3., 10.1374}}	{98.8775, 111.283}	inf	49.	3583.49	{426.032, 4635.72}	7213.25	1.58797	newly	90.	3269.88	{474., 2983.}	3533.2	1.2843	Reff	0.726881	0.50663	{0.0621526, 0.71689}	32.7838	ar	44 237.	164 683.	{156 109., 168 845.}	272.274
43	R0	{1.33756, 2.92653}	{2.56075, 6.96547}	{{2.02608, 5.26972}, {3., 7.6621}}	{91.4497, 138.011}	inf	64.	5437.32	{1066.66, 5253.09}	8395.82	1.53967	newly	97.	5084.24	{686., 5800.}	5144.74	1.28064	Reff	0.706272	0.512067	{0.216006, 1.35834}	68.1872	ar	47 342.	178 824.	{158 759., 228 639.}	277.728
44	R0	{1.35801, 2.92276}	{2.54307, 7.73446}	{{1.88554, 0.706755}, {3., 10.5907}}	{87.2646, 185.883}	inf	37.	1669.39	{252.201, 1242.27}	4411.86	1.38212	newly	67.	1415.52	{133., 1952.}	2012.72	0.996397	Reff	0.707754	0.435136	{0.119254, 0.741091}	60.6978	ar	47 940.	171 527.	{163 781., 167 339.}	257.795
45	R0	{1.31342, 4.02649}	{2.7493, 6.78247}	{{2.15737, 3.38882}, {3., 10.1855}}	{109.323, 84.2831}	inf	207.	1473.1	{300.056, 1452.04}	619.098	0.572404	newly	308.	1283.34	{174., 1209.}	339.234	0.476875	Reff	0.755532	0.673857	{0.30416, 1.17757}	42.7988	ar	42 995.	161 582.	{151 967., 171 544.}	275.816
46	R0	{1.37919, 3.75889}	{2.73871, 13.2568}	{{2.28893, 10.0274}, {3., 15.3601}}	{98.5738, 252.678}	inf	174.	5282.	{465.295, 12 214.8}	2937.23	1.21559	newly	255.	2858.33	{164., 9197.}	1034.75	0.832978	Reff	0.704463	0.750409	{0.0783537, 2.42415}	64.0836	ar	49 157.	179 458.	{158 027., 255 102.}	265.071
47	R0	{1.37458, 3.56117}	{2.80108, 9.48296}	{{2.41213, 0.206887}, {3., 13.9885}}	{103.777, 220.895}	inf	54.	6809.6	{1343.77, 4348.38}	12 511.7	1.8718	newly	75.	9596.01	{2023., 13 376.}	12 696.	1.69566	Reff	0.710232	1.03443	{0.609182, 2.4121}	50.9636	ar	48 478.	187 386.	{163 421., 248 170.}	286.538
48	R0	{1.33393, 3.78296}	{2.45333, 4.39379}	{{1.39094, 0.24615}, {3., 14.9631}}	{83.9172, 98.2577}	inf	157.	2253.45	{1598.75, 3233.63}	1335.32	1.05398	newly	214.	2759.68	{939., 4126.}	1190.09	1.01556	Reff	0.740251	0.595144	{0.206455, 0.647315}	44.7449	ar	44 885.	165 068.	{156 384., 184 233.}	267.759
49	R0	{1.24918, 2.39046}	{2.55712, 13.5495}	{{1.14898, 6.41928}, {3., 17.6048}}	{108.039, 466.817}	inf	26.	5123.24	{774.466, 4961.9}	19 604.8	2.03602	newly	69.	3456.2	{270., 4936.}	4908.99	1.31343	Reff	0.801174	0.618668	{0.325444, 0.847393}	36.4658	ar	35 969.	173 400.	{160 384., 190 719.}	382.082
50	R0	{1.28281, 2.3032}	{2.31644, 10.3049}	{{1.60416, 1.55467}, {3., 16.2135}}	{80.5755, 373.324}	inf	27.	3300.02	{2654.05, 3693.08}	12 122.3	1.98787	newly	59.	5354.95	{957., 10 159.}	8977.81	1.58782	Reff	0.767787	0.618069	{0.163307, 1.60365}	53.9585	ar	40 223.	172 768.	{157 463., 222 224.}	329.525
